# Deciphering risk factors for blood stream infections, bacteria species and antimicrobial resistance profiles among children under five years of age in North-Western Tanzania: a multicentre study in a cascade of referral health care system

**DOI:** 10.1186/s12887-019-1411-0

**Published:** 2019-01-26

**Authors:** J. Seni, A. A. Mwakyoma, F. Mashuda, R. Marando, M. Ahmed, R. DeVinney, J. D. D. Pitout, S. E. Mshana

**Affiliations:** 1 Department of Microbiology and Immunology, Weill-Bugando School of Medicine, Catholic University of Health and Allied Sciences, P.O. Box 1464, Mwanza, Tanzania; 2 Department of Microbiology, Immunology and Infectious Diseases, Cumming School of Medicine, University of Calgary, 3330 Hospital Dr NW, Calgary, AB T2N 4N1 Canada; 3 Department of Paediatrics and Child Health, Bugando Medical Centre, Catholic University of Health and Allied Sciences, P.O. Box 1370 - 1464, Mwanza, Tanzania

**Keywords:** Blood stream infections, Children, Tanzania

## Abstract

**Background:**

Blood stream infections (BSIs) cause a complex cascade of inflammatory events, resulting in significant morbidity and mortality in children in Tanzania. This study was designed to delineate circulating bacterial species, antimicrobial resistance (AMR) profiles and risk factors for BSIs and mortality among children in the cascade of referral health care facilities so as to guide comprehensive BSIs management.

**Methods:**

A multiple cross sectional analytical study was conducted between July 20, 2016 to October 04, 2017 involving 950 children less than five years of age in the North-western part of Tanzania. Children with clinical features suggestive of BSIs were included. Demographic, clinical and laboratory information on culture and antimicrobial susceptibility testing was collected from children; and analyzed using STATA version 13.0 software.

**Results:**

The prevalence of BSIs among children was 14.2% (95% CI: 12.1–16.6%), with specific prevalence in the district, regional and tertiary hospitals being 8.3, 6.4 and 20.0%, respectively. The most common bacterial pathogens isolated from 135 culture-positive children were *Klebsiella pneumoniae* (55, 40.4%), *Staphylococcus aureus* (23, 17.0%), and *Escherichia coli* (17, 12.6%). Multi-drug resistance (MDR) was higher in isolates from children at Bugando Medical Centre (BMC) tertiary hospital than isolates from district and regional hospitals [OR (95% CI): 6.36 (2.15–18.76); *p* = 0.001]. Independent risk factors for BSIs were neonatal period [OR (95% CI): 1.93 (1.07–3.48); *p* = 0.003] and admission at BMC [2.01 (1.08–3.74); *p* = 0.028)]. Approximately 6.6% (61/932) of children died, and risk factors for mortality were found to be children attending BMC [OR (95% CI): 4.95 (1.95–12.5); p = 0.001)], neonatal period [OR (95% CI): 2.25 (1.02–5.00); *p* = 0.045)], and children who had blood culture positive results [OR (95% CI): 1.95 (1.07–3.56); *p* = 0.028)].

**Conclusions:**

The prevalence of BSIs (14.2%) in this multi-centre study is high and predominantly caused by the MDR *K. pneumoniae*. Priority interventional measures to combat BSIs and mortality, specifically among neonates at BMC are urgently recommended.

## Background

Blood stream infections (BSIs) are the most common causes of morbidity and mortality in children [1, 2]. They constitute a complex cascade of inflammatory processes spanning from systemic inflammatory response syndrome, sepsis, severe sepsis, septic shock and ultimately death if not promptly managed [3–5].

Introduction of vaccines and the advancements in technology, with more invasive diagnostic and treatment modalities has resulted in a paradigm shift in both implicated etiological agents as well as the age-groups affected by BSIs [6–8]. As a result, previously dominant bacteria such as *Streptococcus pneumoniae, Haemophilus influenzae* type b and *Neisseria meningitidis*, are currently outnumbered by multidrug resistant (MDR) bacteria like Methicillin resistant *Staphylococcus aureus* (MRSA) and Extended spectrum beta lactamase (ESBL) producing enterobacteriaceae, which in most cases are of nosocomial origin [6–8]. A recent review of ESBL attributable BSIs in children across the world showed varying magnitude across countries, ranging from 10 to 15% (Africa, South America and South-Eastern Asia), and below 5% in Europe [9].

In Tanzania, previous studies which were largely centered in the tertiary health care facilities showed that the proportion of BSIs ranged from 5 to 15%, with ESBL producing *Klebsiella pneumoniae* and *Escherichia coli* being the most predominant pathogens [10–14]. In this regard, findings from these studies cannot be generalized to all levels of health care facilities in Tanzania [10–14]. Of note, mortality in these studies was unacceptably high (in some studies up to 20%), calling for interventional measures in these tertiary hospitals, along with evaluating the trend in other health care facilities like regional/referral and district hospitals.

This study evaluated the magnitude of BSIs, bacterial species, and antimicrobial resistance (AMR) profiles among children attending different health care facilities in the North-western part of Tanzania to guide specific antimicrobial therapies. Moreover, risk factors for BSIs and mortality were ascertained so as inform specific target groups for preventive and control measures.

## Methods

### Study design and settings

**Fig. 1:**
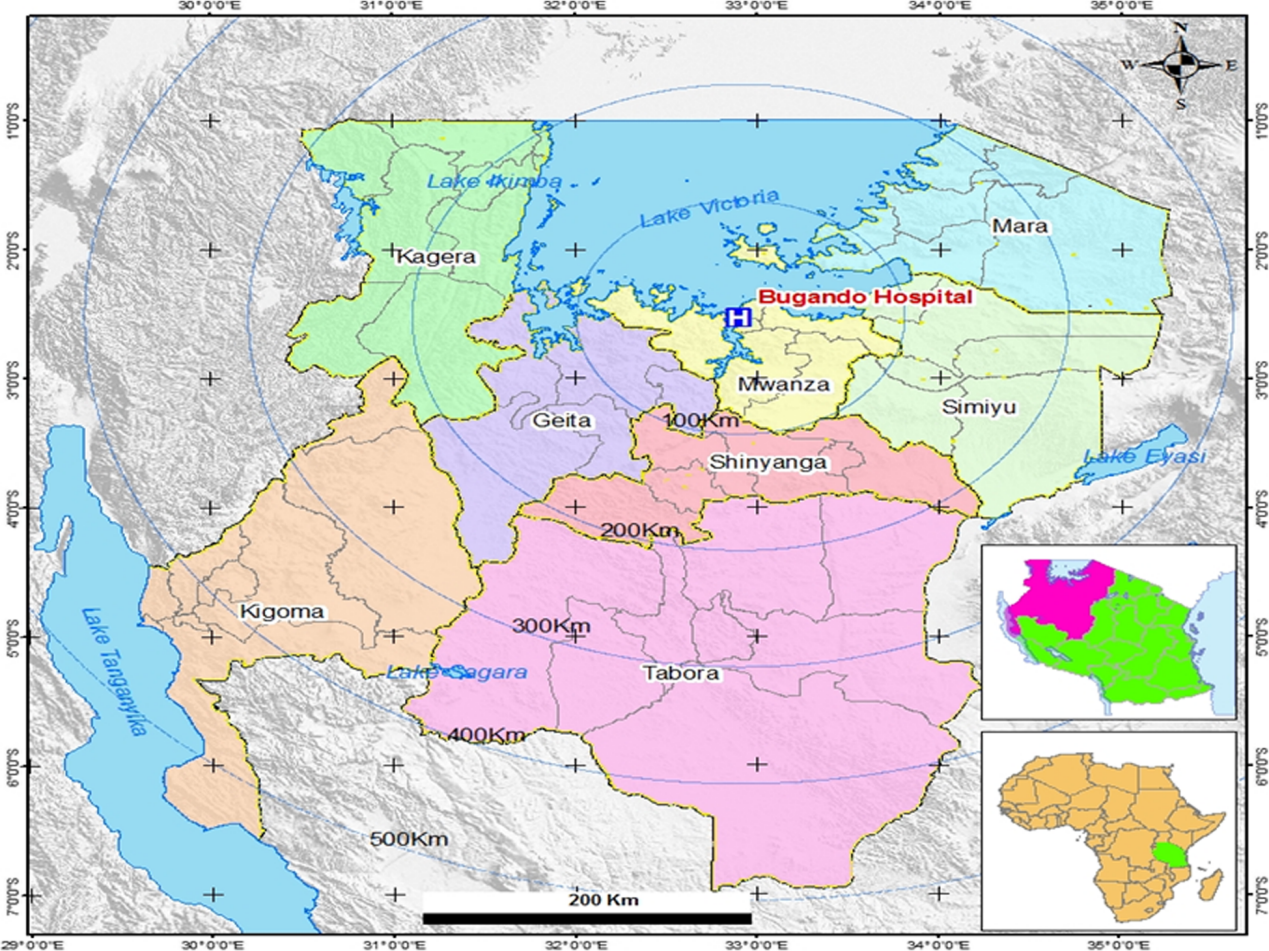
The map showing North-western part of Tanzania. Africa and Tanzania maps (inserts); Area marked in apple green in the Africa map is Tanzania; Area marked in pink in the Tanzania map is the catchment area for the study in the North-western part of Tanzania. Bugando Medical Centre (a tertiary hospital) and eight administrative regions forming its catchment area are labeled. This map was produced using the base map obtained from the Tanzanian Land Survey Department [48], using Quantum Geographic Information System (Quantum GIS), a software for mapping [49]

This was a multiple cross sectional analytical study conducted from July 20, 2016 to October 04, 2017 involving four health care facilities in the cascade of referral system in North-western Tanzania. These health care facilities were Bugando Medical Centre (BMC), a tertiary hospital, Sekou Toure Regional Referral Hospital (SRRH), Nyamagana District Hospital (NDH) to represent an urban setting, and Sengerema District Designated Hospital (SDDH) to represent a rural setting. All these health care facilities are teaching hospitals for the Catholic University of Health and Allied Sciences (CUHAS), except NDH (Table 1 and Fig. 1).

**Table 1:** Demographic descriptions of health facilities involved and respective number of children enrolled

Level/rank of HCF	HCF involved	HCF catchment population	HCF bed capacity	Study participants enrolled (%)
Tertiary	BMC (urban)	16,252,410	950	514 (54.1)
Regional/referral	SRRH (urban)	2,772,509	375	218 (23.0)
District	NDH (urban)	363,452	88	80 (8.4)
	SDDH (rural)	663,034	320	138 (14.5)

Sources: Hospital Records; Tanzania Population and Health Census (2012) and Staffing Levels for Ministry of Health Tanzania (2014–2019). HCF: Health care facility; BMC: Bugando Medical Center; SRRH: Sekou Toure Regional Hospital; NDH: Nyamagana District Hospital; SDDH: Sengerema District Designated Hospital

Ideal bed capacity in health care facilities in Tanzania are 550 to 1500 beds for tertiary hospitals; 176 to 450 beds for regional referral hospitals; and 150 to 175 beds for district hospitals

### Study population, inclusion and exclusion criteria

The study enrolled prospectively children presenting to the health care facilities with clinical symptoms and signs suggestive of BSIs [5, 13], and whose parents/guardians voluntarily consented to participate on their behalf. The clinical signs and symptoms for enrollment were based on the WHO Young Infant Study Group and its methodology paper i.e. temperature (of > 38 °C or < 36 °C), age specific tachycardia, age specific tachypnoea, convulsions, altered state of consciousness and abnormal feeding [5]. To ensure consistency, enrolment evaluation was done by paediatrician and/or experienced registrar who were also part of this study. A sample size was estimated by the Kish Leslie formula, using previous prevalence of BSIs among children of 7.4% in Mwanza. This resulted into a minimum of 106 children per site and 424 children in all four sites [15]. Taking into account different hospital bed capacities, a total of 1008 children under 5 years of age were prospectively enrolled during the study period. Fifty eight (5.7%) children were excluded because of incomplete information in the questionnaires and/or medical records. Also, using unique identifying numbers, children who were already enrolled in the lower level health care facilities and referred to another heath care facility which was also a study site were excluded. Therefore, this resulted into a total of 950 children under 5 years (Table 1). This sample size sufficed to estimate the primary study end-points (i.e. the overall prevalence and health facility-level specific prevalence of BSIs, bacterial species and AMR profiles), and the study secondary end-points (risk factors for BSIs and mortality).

### Data collection and laboratory procedures

Socio-demographic and clinical characteristics of children were collected using a structured pre-tested questionnaire. Absolute age of children (in months) was collected and then during analysis, three key groups were delineated i.e. neonates (≤ 1 month), infants (2 to 12 months) and other children (13 to 60 months). Moreover, clinical information like co-morbidities such as HIV infection, malnutrition, sickle cell disease, pneumonia, anemia and congenital anomalies (to mention a few) were obtained from patient medical records. Additionally, calculation of body weight was done to categorize children into normal weight (z-score between 2 and − 2); underweight (z-score between − 2 and − 3) or overweight (z-score between 2 and 3) for the respective age using the WHO Child Growth Standards for boys and girls [http://www.who.int/childgrowth/standards/cht_wfa_girls_p_0_5.pdf?ua=1 and http://www.who.int/childgrowth/standards/cht_wfa_boys_p_ 0_5.pdf?ua=1].

The Tanzania Algorithm for HIV testing among children above 18 months of age employs SD Bioline HIV 1/2 test (Standard Diagnostics Inc., California, USA) as the first test, and if reactive, it is confirmed by a second serological test, the Unigold HIV test (Trinity Biotech, Bray, Ireland). For children below 18 months of age HIV diagnosis is done by HIV DNA PCR [16, 17].

About two to five milliliters of blood sample from each child was collected and inoculated into Brain Heart Infusion broth (OXOID, UK) in a ratio of blood to Brain Heart Infusion of 1:10. The samples from SDDH were analysed at SDDH Laboratory, whereas samples from the rest of the study sites were analysed at the CUHAS Multipurpose Laboratory as previously described [18, 19].

AST was done by the conventional Kirby–Bauer disk diffusion method using the Clinical Laboratory Standard Institute guidelines [20]. The phenotypic screening of ESBL was done in Muller Hinto agar (OXOID, UK) along with other disks, using a cut-off zone inhibition of ≤25 mm for ceftriaxone and ≤ 22 mm for ceftazidime [20]. Confirmation of ESBL production among *E.coli*, *K. pneumoniae,* and *Proteus mirabilis* was done in Muller Hinton agar by double disc synergy method [21]. MRSA was confirmed by the use of cefoxitin disc (30 μg) and strains showing zone of inhibition of ≤21 mm were labelled as MRSA [20]. A bacterial strain was confirmed to be MDR when it was resistant to at least one agent in three or more classes of antimicrobial agents [22]. *E. coli* ATCC 25922 and *Staphylococcus aureus* ATCC 25923 were used as reference strains for Gram negative and Gram positive bacteria, respectively in quality control of culture media, biochemical identification tests and AST.

### Data management

Data were analyzed by the STATA version 13.0 software (College Station, Texas, USA). Proportions of children with culture-confirmed BSIs, bacterial species, and resistance to various antimicrobial agents were determined. Univariate logistic regression analysis was done to all variables, but only variables with a *p*-value of less than 0.05 were subjected to multivariate logistic regression analysis. Independent risk factors for BSIs and mortality among children were determined by multivariate logistic regression analysis using odds ratios, 95% confidence intervals and p-value cut-off of less than 0.05.

## Results

### Socio-demographic and clinical characteristics of children enrolled

The median age (IQR) of the participants was 9 (1–23) months, with minimum and maximum age being less than 1 month and 60 months, respectively. The most common age group was children above 1 year of age, 41.6% (*n* = 395); followed by neonates, 36.4% (*n* = 346). The median weight (IQR) for different age categories were: neonates [2.9 (2.5–3.4) kg], children between 2 to 12 months [7.5 (5.5–8.5) kg] and children above 1 year of age [10.7 (9.0–13.0) kg]. A total of 392 (41.3%) children had underlying co-morbidities and the majority of children presented with fever, 86.2% (*n* = 819) (Table 2). Of the 950 children enrolled, the proportions of specific co-morbidities were malnutrition (13.2%), prematurity (5.3%), HIV (3.9%), and sickle cell disease (3.1%).

**Table 2:** Socio-demographic and clinical characteristics of children

Characteristic		Number (%)
Sex	Boys	558 (58.7)
	Girls	392 (41.3)
Age group	≤ 1 month	346 (36.4)
	2–12 months	209 (22.0)
	13–60 months	395 (41.6)
Weight (kg)^a^	Normal	539 (56.7)
	Underweight	373 (39.3)
	Overweight	38 (4.0)
Residence	Rural	315 (33.2)
	Urban	635 (66.8)
Mwanza city	No	313 (33.0)
	Yes	637 (67.0)
Current antibiotic use	No	545 (57.4)
	Yes	405 (42.6)
History of admission in the last 3 months^b^	No	525 (86.9)
	Yes	79 (13.1)
Presence of i/v line	No	254 (26.7)
	Yes	696 (73.3)
Presence of indwelling urinary catheter	No	930 (97.9)
	Yes	20 (2.1)
Co-morbidities^c^	No	558 (58.7)
	Yes	392 (41.3)
Presenting symptoms and signs		
Fever	Yes	819 (86.2)
Tachypnoea	Yes	235 (24.7)
Tachycardia	Yes	186 (19.6)
Convulsions	Yes	123 (13.0)
Loss of consciousness	Yes	14 (1.5)

^a^ Weight adjusted to age; ^b^ Non neonates; ^c^ Malnutrition (*n* = 105), Respiratory tract infections (*n* = 86), Prematurity (*n* = 50), Congenital anomalies (*n* = 36: congenital heart diseases, neural tube defects, hydrocephalus and others), Anemia (*n* = 31), Sickle cell disease (*n* = 26), HIV (*n* = 19), Skin and soft tissue infections (*n* = 8); Necrotizing enterocolitis (*n* = 3); Amoebiasis (*n* = 2); Burn injury (*n* = 2); Rheumatic heart diseases (*n* = 2); Cerebral malaria (*n* = 1); Spinal injury (*n* = 1); Malnutrition and HIV (*n* = 17); Malnutrition and sickle cell disease (*n* = 3); Premature and HIV (*n* = 1)

### Prevalence of blood stream infections among children in North-Western Tanzania

**Fig. 2:**
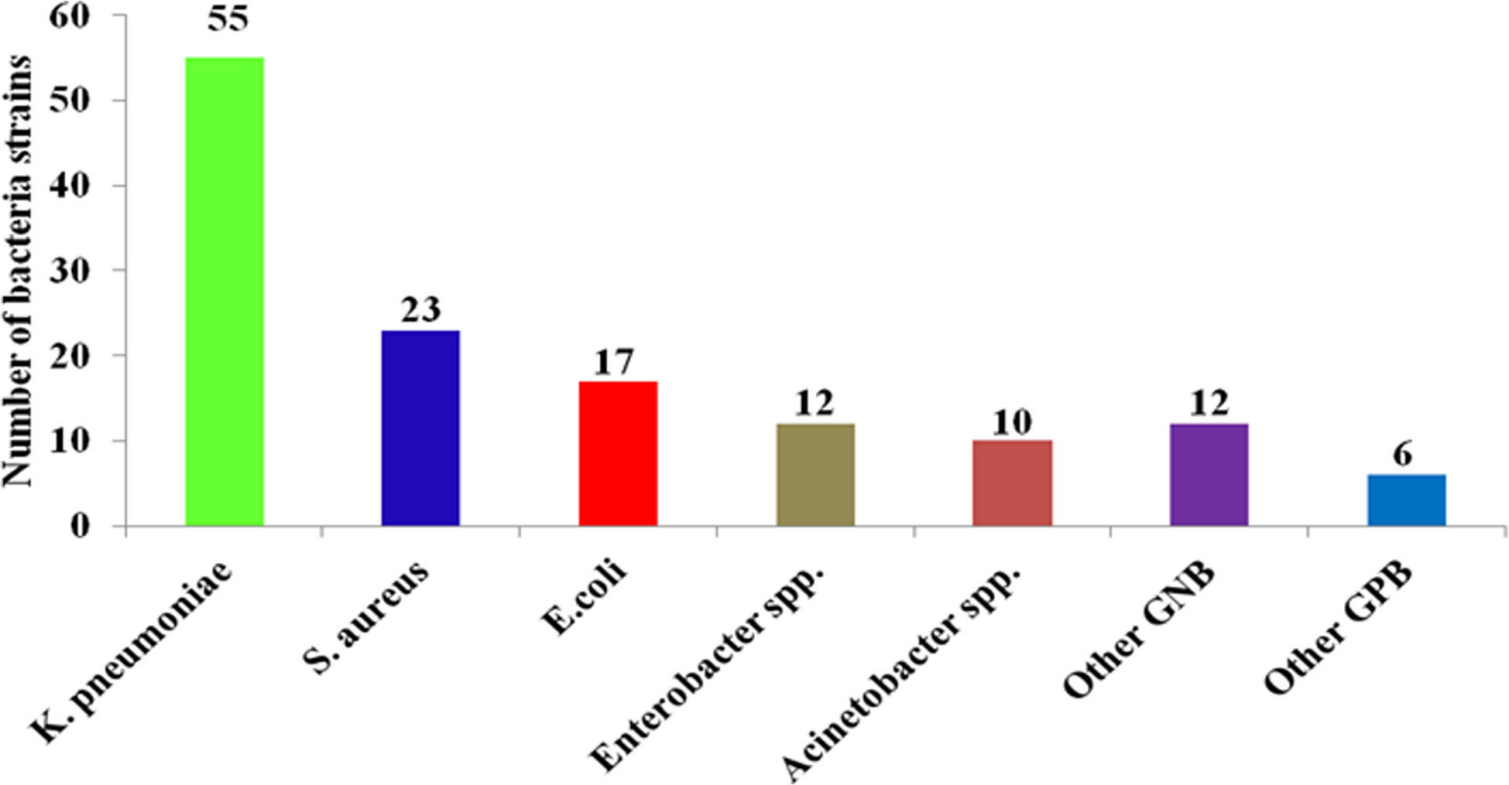
Bacteria species strains from children with blood stream infections. Other Gram negative bacteria (GNB): *Citrobacter freundii (5), Salmonella spp. (1); Serratia marcescens (1); Morganella morganii (1); Pseudomonas aeruginosa (1), Chromobacterium violaceum (1), unidentified GNB (2).* Other Gram positive bacteria (GPB): *Enterococcus spp. (3), Streptococcus pyogenes (1) and other Streptococcus spp. (2)*

The prevalence of BSIs among children was 14.2% (95%CI: 12.1–16.6%), with specific prevalence in the district, regional and tertiary hospitals being 8.3, 6.4 and 20.0%, respectively. Also, the age-specific prevalence of BSIs for neonates, children between 2 to 12 months and children above 12 months were 25.4% (88/346), 5.7% (12/209) and 8.9% (35/395), respectively. The most common bacteria species were *K. pneumoniae* (55, 40.4%), *S. aureus* (23, 17.0%), and *E. coli* (17, 12.6%). There was an overall preponderance of BSIs with Gram negative bacteria (78.5%) compared to BSIs attributable to Gram positive bacteria (21.5%); *p* < 0.001 (Fig. 2).

### Antimicrobial resistance patterns of bacteria causing blood stream infections

The majority of bacteria were resistant to ampicilllin and trimethoprim-sulfamethoxazole with resistance rates ranging from 66.6 to 100.0%. All Gram negative bacteria were sensitive to meropenem, except one *Acinetobacter spp* isolate. The resistance of *Acinetobacter spp.* to piperacillin and piperacillin-tazobactam was 100 and 50.0%, respectively. One *Pseudomonas aeruginosa* isolate was resistant to piperacillin and ceftazidime, but sensitive to gentamicin, ciprofloxacin, piperacillin-tazobactam and meropenem. The third generation cephalosporin resistance (3rd gen Ceph-R) was strikingly high in *K. pneumoniae* (95.7%), *E. coli* (58.8%), and other Gram negative Enterobacteriaceae (69.6%). *E. coli* and *K. pneumoniae* strains which were 3rd gen Ceph-R were all confirmed to be ESBL producers. The proportion of MRSA among *S. aureus* strains was 34.7%. The distributions of eight MRSA strains in children with BSIs in health care facilities were: two in NDH & SDDH, two in SRRH and four in BMC, nevertheless this distribution was not statistically significant (*p* = 0.510). Two MRSA strains (8.7%) were found to be non-susceptible to vancomycin (Table 3).

**Table 3:** Antimicrobial resistance patterns of bacteria causing blood stream infections

Bacteria (n)	Antimicrobial resistance (%)									
	AMP	SXT	GEN	CIP	ERY	VAN	AMC	CAZ	CRO	MER
*K. pneumoniae* (55)	100	96.3	78.2	29.1	NA	NA	94.6	90.9	95.7	0.0
*S. aureus* (23)	100	82.6	26.1	34.8	65.2	13.0	NA	NA	NA	NA
*E. coli* (17)	100	94.1	40.1	52.9	NA	NA	94.1	52.9	58.8	0.0
*Acinetobacter* spp. (10)	NA	90.0	40.0	10.0	NA	NA	NA	100	NA	10.0
Other GNB (23)	95.6	78.3	60.9	17.4	NA	NA	82.6	60.6	69.6	0.0
Other GPB (6)	83.3	66.7	33.3	50.0	50.0	0.0	NA	NA	NA	NA

*AMP* Ampicillin, *SXT* Trimethoprim-sulfamethoxazole, *GEN* Gentamicin, *CIP* Ciprofloxacin, *ERY* Erythromycin, *VAN* Vancomycin, *AMC* Amoxycillin-clavulanate, *CRO* Ceftriaxone, *CAZ* Ceftazidime, *MEM* Meropenem, *NA* Not applicable*.* Other Gram negative bacteria (GNB): *Enterobacter spp. (12), Citrobacter freundii (5), Salmonella spp. (1); Serratia marcescens (1); Morganella morganii (1); Chromobacterium violaceum (1), Unidentified GNB (2).* Other Gram positive bacteria (GPB): *Enterococcus spp. (3), Streptococcus pyogenes (1*) *and other Streptococcus spp*. *(2)*

### Cephalosporin resistant and multi-drug resistant bacterial strains attributable blood stream infections

The overall proportion of 3rd gen Ceph-R among members of the family Enterobacteriaceae was 79.0% (75/95). Irrespective of the bacteria species, 3rd gen Ceph-R was significantly higher in isolates from BMC tertiary hospital [OR (95%CI): 4.95 (1.15–21.32); *p* = 0.032], than those from district and regional hospitals (Table 4).

**Table 4:** Cephalosporin resistance among Enterobacteriaceae causing blood stream infections

Health facility (N)	Cephalosporin resistant strains attributable blood stream infections		
	(n, %)	OR (95%CI)	*p*-value
NDH & SDDH (9)	5 (55.6)	1	
SRRH (7)	2 (28.6)	0.32 (0.04–2.62)	0.288
BMC (79)	68 (86.1)	4.95 (1.15–21.32)	0.032
Total (95)	75 (79.0)		

Screening for Ceph-R was done to all Gram negative bacteria belonging to the family Enterobacteriaceae; BMC: Bugando Medical Center; SRRH: Sekou Toure Regional Referral Hospital; NDH: Nyamagana District Hospital; SDDH: Sengerema District Designated Hospital

Over three quarters of bacteria strains were found to be MDR [77.8% (105/135)], with the majority of these being Gram negative bacteria compared to Gram positive bacteria [81.9% (86/105) versus 18.1% (19/105), *p* < 0.001]. The distribution of MDR among isolates from children with BSIs in tertiary hospital, regional/referral hospital and two district hospitals were 86.4% (89/103), 50.0% (7/14) and 50.0% (9/18), respectively. MDR was significantly higher in strains from BMC tertiary hospital [OR (95% CI): 6.36 (2.15–18.76); *p* = 0.001], than those from district and regional hospitals.

### Risk factors of blood stream infections among children in North-Western Tanzania

Children under 5 years of age with low median weight were significantly more associated with BSIs compared to those with higher median weight [3.4 (2.5–8.0) kg versus 7.5 (3.3–10.0) kg*;* p < 0.001]. But when weight was adjusted to age, there was no significant difference between under-weight and overweight children, compared to those with normal weight (Table 5). Other factors which were associated with BSIs on univariate analysis were children admitted at BMC tertiary hospital, neonates, previous use of antibiotics, prematurity and malnutrition. On multivariate logistic regression analysis, neonatal period and admission at BMC were found to be the independent risk factors of BSIs [OR (95% CI): 1.93 (1.07–3.48); *p* = 0.003 and 2.01(1.08–3.74); *p* = 0.028), respectively] (Table 5).

**Table 5:** Risk factors of blood stream infections among children in North-western Tanzania

Variable		BSIs (n, %)	Univariate OR (95%CI)	*p*-value	Multivariate OR (95%CI)	*p*-value
Hospital	NDH & SDDH (218)	18 (8.3)	1			
	SRRH (218)	14 (6.4)	0.76 (0.37–1.57)	0.464	0.90 (0.43–1.90)	0.917
	BMC (514)	103 (20.0)	2.78 (1.64–4.72)	< 0.001	2.01 (1.08–3.74)	0.028
Sex	Boys (558)	80 (14.3)	1			
	Girls (392)	55 (14.0)	0.97 (0.67–1.41)	0.894		
Age category	13–60 months (395)	35 (8.9)	1			
	2–12 months (209)	12 (5.7)	0.63 (0.32–1.23)	0.177	0.59 (0.30–1.17)	0.128
	≤1 month (346)	88 (25.4)	3.51 (2.30–5.36)	< 0.001	1.93 (1.07–3.48)	0.030
Weight (kg)	Normal (539)	85 (15.8)	1			
	Underweight (373)	49 (13.1)	0.81 (0.55–1.18)	0.270		
	Overweight (38)	1 (2.6)	0.14 (0.02–1.07)	0.058		
Residence	Rural (315)	39 (12.4)	1			
	Urban (635)	96 (15.1)	1.26 (0.85–1.88)	0.256		
Mwanza city	No (313)	41 (13.1)	1			
	Yes (637)	94 (14.8)	1.15 (0.78–1.71)	0.492		
Current antibiotic use	No (545)	66 (12.1)	1			
	Yes (405)	69 (17.0)	1.49 (1.03–2.15)	0.032	1.42 (0.97–2.08)	0.069
Previous admission*	No (525)	39 (7.4)	1			
	Yes (79)	8 (10.1)	1.40 (0.63–3.13)	0.406		
Intravenous line	No (254)	27 (10.6)	1			
	Yes (696)	108 (15.5)	1.54 (0.99–2.42)	0.058		
Urinary catheter	No (930)	132 (14.2)	1			
	Yes (20)	3 (15.0)	1.07 (0.31–3.69)	0.919		
Co-morbidities**	No (558)	85 (15.2)	1			
	Yes (392)	50 (12.8)	0.81 (0.56–1.18)	0.282		
Prematurity	No (900)	121 (13.4)	1			
	Yes (50)	14 (28.0)	2.50 (1.31–4.78)	0.005	1.15 (0.58–2.25)	0.691
Malnutrition	No (825)	126 (15.3)	1			
	Yes (125)	9 (7.2)	0.43 (0.21–0.87)	0.019	0.52 (0.22–1.20)	0.126
HIV	Negative (913)	129 (14.1)	1			
	Positive (37)	6 (16.2)	1.18 (0.48–2.88)	0.722		
SCD	No (921)	132 (14.3)	1			
	Yes (29)	3 (10.3)	0.69 (0.21–2.31)	0.547		

*BMC* Bugando Medical Center, *SRRH* Sekou Toure Regional Referral Hospital, *NDH* Nyamagana District Hospital, *SDDH* Sengerema District Designated Hospital:* In the past three months (excluding current admission); **Malnutrition (n = 105), Respiratory tract infections (n = 86), Prematurity (n = 50), Congenital anomalies (n = 36: congenital heart diseases, neural tube defects, hydrocephalus and others), Anemia (n = 31), SCD: Sickle cell disease (n = 26),HIV (n = 19), Skin and soft tissue infections (n = 8); Necrotising enterocolitis (n = 3); Amoebiasis (n = 2); Burn injury (n = 2); Rheumatic heart diseases (n = 2); Cerebral malaria (n = 1); Spinal injury (n = 1); Malnutrition and HIV (n = 17); Malnutrition and sickle cell disease (n = 3); Premature and HIV (n = 1)

### Management outcomes among children with blood stream infections

Out of 950 children, 18 (1.9%) could not be followed to the end because they were referred to other hospitals and their respective information could not be traced. Of the remaining 932 children, 871 (93.4%) were treated successfully and discharged, and unfortunately 61 (6.6%) died. The median length of hospital stay (IQR) was 5 (3–10) days, minimum and maximum of 1 day and 70 days, respectively. The median length of hospital stay (IQR) was longer among children who were culture positive [7 (3–14) days] compared to those who were culture negative [4 (2–9) days] (*p* < 0.001). Bacteria species-specific mortality was: *K. pneumonieae* (14.8%, 8/54), *E. coli* (23.5%, 4/17), *S. aureus* (4.4%, 1/23), *Acinetobacter* spp. (9.1%, 1/9), Other GNB (22.7%, 5/22) and other GPB (16.7%, 1/6). Moreover, out of eight children who had MRSA attributable BSIs, one (12.5%) died.

On univariate analysis, more children with 3rd gen Ceph-R died compared to those with non-3rd gen Ceph-R [18.7% versus 16.7%, *p* = 0.844]. Also, more children with MDR attributable BSIs died compared to non-MDR BSIs [16.4% versus 10.3%, *p* = 0.428], although the difference was not statistically significant. On multivariate logistic regression analysis, the independent risk factors for mortality were found to be children attending BMC [OR (95% CI): 4.95 (1.95–12.5); *p* = 0.001)], neonatal period [OR (95% CI): 2.25 (1.02–5.00); *p* = 0.045)], and children who had blood culture positive results [OR (95% CI): 1.95 (1.07–3.56); *p* = 0.028)] (Table 6).

**Table 6:** Risk factors of mortality among children with blood stream infections

Variable		Deaths (n, %)	Univariate OR (95%CI)	p-value	Multivariate OR (95%CI)	*p*-value
Hospital	NDH & SDDH (217)	2 (0.9)	1			
	SRRH (203)	0 (0.0)	–		–	
	BMC (512)	59 (11.5)	14.0 (3.39–57.84)	< 0.001	4.95 (1.95–12.5)	0.001
Sex	Boys (548)	43 (7.9)	1			
	Girls (384)	18 (4.7)	0.58 (0.33–1.02)	0.058		
Age category	13–60 months (386)	9 (2.3)	1			
	2–12 months (203)	7 (3.5)	1.50 (0.55–4.08)	0.431	1.32 (0.48–3.66)	0.592
	≤1 month (343)	45 (13.1)	6.33 (3.04–13.15)	< 0.001	2.25 (1.02–5.00)	0.045
Residence	Rural (308)	17 (5.5)	1			
	Urban (624)	44 (7.1)	1.30 (0.73–2.31)	0.375		
Mwanza city	No (307)	18 (5.86)	1			
	Yes (625)	43 (6.88)	1.19 (0.67–2.09)	0.556		
Co-morbidities	No (549)	35 (6.4)	1			
	Yes (383)	26 (6.8)	1.07 (0.63–1.81)	0.802		
Prematurity	No (883)	51 (5.8)	1			
	Yes (49)	10 (20.4)	4.18 (1.98–8.86)	< 0.001	1.70 (0.77–3.73)	0.186
Culture	Negative (799)	41 (5.1)	1			
	Positive (133)	20 (15.0)	3.27 (1.85–5.79)	< 0.001	1.95 (1.07–3.56)	0.028
3rd gen. Ceph-R	No (18)	3 (16.7)	1			
	Yes (75)	14 (18.7)	1.15 (0.29–4.51)	0.844		
MDR	No (29)	3 (10.3)	1			
	Yes (104)	17 (16.4)	1.69 (0.46–6.23)	0.428		

*BMC* Bugando Medical Center, *SRRH* Sekou Toure Regional Referral Hospital, *NDH* Nyamagana District Hospital, *SDDH* Sengerema District Designated Hospital; 3rd gen. *Ceph-R* Third generation cephalosporin resistance, *MDR* Multi-drug resistance

## Discussion

### The magnitude of blood stream infections and bacteria pathogens among children

This current large multi-centre study has shown a higher prevalence of children with BSIs (14.2%), compared to two previous studies in the general pediatric population in the same region (6.6 and 7.4%), and other countries like Malawi (7.5%), Cambodia (9.1%), in six countries across the world (10.6%), Spain and the USA (< 1.5%) [13, 15, 23–28]. Our results are comparable to another previous study in the same region among malnourished children (13.9%) [14]. Similar to the current study, a review of BSIs in developing countries and other previous studies in Dar es Salaam and Kilimanjaro, Tanzania reported that more than half of children get BSIs due to *S. aureus*, *E. coli* and *Klebsiella* spp. (range: 39 to 70%) [11, 29, 30]. However, in the current study the most common bacteria species was *K. pneumoniae.* The study in Kilimanjaro showed that nearly a quarter of pathogens implicated were *Salmonella enterica* [11]. The difference can be accounted for by the high prevalence of HIV infections among children enrolled in the study in Kilimanjaro (12.2%), as opposed to 3.9% in the current study. It is well known that HIV/AIDS is an important risk factor for invasive salmonellosis in both children and adult febrile patients [11, 31]. Three previous studies in Kenya, Uganda and Malawi have also shown similar findings of a predominance of *Salmonella enterica* and its association with HIV infections among children [28, 32, 33]. In most developed countries there is low prevalence of BSIs which is largely related to the high vaccine coverage, stringent IPC and antimicrobial stewardship measures. In these countries, Gram positive bacteria causing BSIs predominate among healthy children [8], whereas *Salmonella enterica* predominate in children with underlying risk conditions like sickle cell disease [26, 27, 34, 35]. On the other hand, low prevalence in a few studies in Tanzania and other LMICs may be due to previous use of antibiotics before admission which in turn lead to culture negative results in the majority of non-neonatal children with community on-set BSIs or improved IPC measures in some hospitals.

### Antimicrobial resistance profiles of bacteria causing blood stream infections

The proportion of 3rd gen Ceph-R among members of the family Enterobacteriaceae in the current study is alarmingly higher (79.0%) than the 25 to 50% reported before in the same region, and is predominated by *K. pneumoniae* [14, 19]. All Gram negative bacteria were sensitive to meropenem, except one *Acinetobacter* spp. High AMR among Gram negative bacteria is similar to a previous report involving six countries in Africa, Asia and South America: gentamicin (43%), ciprofloxacin (35%), 3rd gen Ceph (61.3%) and meropenem (11.1%) [24]. The predominance of MDR *K. pneumoniae* compared to *E. coli* has also been reported in an extensive review from developing countries [30]. The majority of Gram positive bacteria were sensitive to vancomycin, and over two third were sensitive to gentamicin. The proportion of MRSA among *S. aureus* strains in the current study is higher (34.7%), than the 28.0% in Mwanza and 23.3% in Dar es salaam reported 8 years ago [19, 36]. As a result, there is an urgent need to introduce routine culture and AST in hospitals lacking this service for all children with clinical features suggestive of BSIs to ensure rational antimicrobial therapies. This is especially important as the remaining antimicrobial therapeutic options like meropenem for Gram negative bacteria, and vancomycin for Gram positive bacteria are very expensive, and have adverse effects in children if not monitored carefully [37–39]. The findings of AMR profiles in different health care facilities in North-western Tanzania are pivotal in addressing the WHO global action plan to combat AMR in the context of a recently launched National Action Plan on AMR (2017–2022) in the United Republic of Tanzania [40, 41]. Indeed, these findings can be used as baseline data to inform interventional measures, and for future monitoring of AMR trends in different levels of health care facilities in Tanzania.

### Risk factors for blood stream infections among children

The main two added values of the current study is the fact that it was a multi-centre study involving four hospitals in the cascade of referral system in North western Tanzania, and also involved all children under 5 years of age, contrary to other previous studies in this country which were single-centred, and often involving neonates only [12, 19, 29]. In this regard, it allowed stratification of the burden of BSIs in different ranks of health care facilities, and across various age-groups. Children in the neonatal period (odds ratio = 1.93) and those admitted at BMC (odds ratio = 2.10) had increased odds of having BSIs, as opposed to other age-groups and children admitted in other hospitals. Moreover, those admitted in BMC tertiary hospital had 4.96 odds of developing 3rd gen Ceph-R attributable BSIs as opposed to those in the regional and district hospitals (and predominantly by *K. pneumoniae*). Similarly, a study in England and Wales showed 10-fold increase in BSIs among infants as opposed to older children, and also more common in boys than girls [8]. These findings have critical treatment values and policy implications in terms of where stringent screening criteria for BSIs and more resources should be directed as previously described in a state-of-the-art review on current aspects in treatment of sepsis [7].

Other risk factors for BSIs found in this study on univariate analysis were prematurity, unadjusted low median weight and previous exposure to antibiotics. Similarly, earlier studies in East Africa have shown that previous exposure to antibiotics and co-morbidities such as malnutrition, HIV, malaria and anemia were associated with BSIs [11, 13, 14, 28, 32]. Co-existence of malaria in the same area, which is also a febrile illness like BSIs may pose diagnostic and therapeutic challenges [13, 15, 28, 29, 32], and calls for laboratory guided management to ensure favourable treatment outcomes in children [25]. The current study did not find an association between BSIs and invasive procedures such as intravenous lines and urinary catheterization, but a previous study in the USA ascertained the association between central venous lines and BSIs among children with sickle cell disease [26]. Therefore, these predictors should be important factors in raising awareness amongst attending clinicians to take timely blood samples and judiciously start empirical antimicrobial therapies to prevent negative heath impacts, including mortality.

### Management outcomes among children with blood stream infections

The present study showed that the overall mortality was 6.6%, with neonates from BMC tertiary hospital being the most vulnerable age-group in over three quarter of these deaths. This mortality is higher than 1.1% reported from Spain among healthy children [27], but similar to previous studies in eight European countries, six countries in three continents and in Kilimanjaro, Tanzania [11, 24, 35]. However, this mortality is low compared to 13.9 to 34.9% previously reported in four studies in Mwanza and Dar es salaam between 2005 and 2013 [12, 14, 19, 29]. The reason behind low mortality in the current study may be partly due to improved IPC in these hospitals. The differences in mortality reiterate the fact that, neonates and children with underlying co-morbidities like malnutrition and prematurity should be priority target groups for interventional measures against BSIs. Additionally, the preponderance of BSIs attributable deaths among children at BMC may be related to the fact that this hospital takes care of critically ill children as well as children with underlying risky conditions who are referred from other health care facilities for tertiary care.

In Tanzania, a combination of ampiclox and gentamicin (first line treatment) and cefotaxime and gentamicin (second line) are antimicrobial therapeutic options [42]. These therapeutic options were compared in a previous randomised controlled trial in Malawi, and it was found that, a combination of penicillin and gentamicin had similar treatment outcomes compared to ceftriaxone (13.7% versus 16.5% mortality) and both combinations were shown to be safe for infants [43]. But given the rapidly increasing AMR in the present study and a recent report from Malawi (15), laboratory guided antimicrobial therapies should be an enduring next step to ensure good management outcomes among children with BSIs.

Preventive measures for children with BSIs require identification of potential sources of pathogens, and especially the MDR pathogens. In a previous study in our research group, we reported higher ESBL gastrointestinal carriage among delivering mothers (15%) and their newborns (25.4%), with acquisition among neonates occurring predominantly in the first twenty four hours of life [44]. This was higher than 2.9% reported among pregnant women in Norway, but of note, four out of 14 women who remained positive for ESBL strains at delivery transmitted these strains to their newborns as shown by the PFGE analysis of the five mother-neonate pairs [44, 45]. Our recent study at BMC found that, 10.5% of 304 neonates had ESBL-attributable sepsis, and these infections were predicted by admission to the intensive care unit and positive ESBL gastrointestinal carriage by mothers and neonates [46]. This was also higher than the 2.8% reported previously in the USA, connoting possible differences in the IPC measures between these two countries [46, 47]. In both studies the *bla*_CTX-M-15_ gene predominated, and similar strains involved in colonization were found to cause subsequent invasive infections in neonates. However, the predominant strains involved were *K. pneumoniae* ST45 in Tanzania and *E. coli* ST131 in the USA [46, 47]. Therefore, similar delineation of potential sources and dynamics of transmission using genomic approaches is urgently required in other hospitals so as to have a comprehensive interventional strategy in North-western Tanzania.

## Conclusions

The prevalence of BSIs (14.2%) in this multi-centre study among children under 5 years of age in North-western Tanzania is comparable to previously reported studies in developing countries, but higher than studies from developed countries. Multidrug resistant *K. pneumoniae* is the predominant pathogen in approximately half of the patients. The overall mortality was 6.6%, with neonates remaining the most vulnerable age-group in over three quarter of these deaths. Strengthening of provision of routine culture and AST services among children with clinical symptoms suggestive of BSIs at BMC tertiary hospital, and introduction of these tests routinely in district and regional hospitals is recommended. Neonates at BMC tertiary hospital should be a specific target group for preventive measures against BSIs.

## Abbreviations

3rd gen Ceph-R: Third generation cephalosporin resistance
AMR: Antimicrobial resistance
AST: Antimicrobial susceptibility testing
BMC: Bugando Medical Centre
BSIs: Blood stream infections
CUHAS: Catholic University of Health and Allied Sciences
ESBL: Extended spectrum beta lactamases
IPC: Infection prevention and control
LMICs: Low and middle income countries
MDR: Multi-drug resistance
MRSA: Methicillin resistant *Staphylococcus aureus*
NDH: Nyamagana District Hospital
SDDH: Sengerema District Designated Hospital
SRRH: Sekou Toure Regional Hospital
